# The Ex-Service Welfare Society

**Published:** 1921-03-05

**Authors:** 


### The Ex-Service Welfare Society.

The cases of shell-shock and neurasthenia which the war has left, form one of the most trying problems which those who have the good of our ex-soldiers at heart try to solve. To aid in this a society has been formed—the Ex-Services Welfare Society—which hopes to establish hostels, not large, but pleasantly situated and adequately equipped for the treatment of such cases. Already a suitable house has been secured in a pleasant part of Putney, where there is a good garden within the walls, and which is near the wide green spaces of Putney Heath. It is hoped to open this hostel in March. To make the Society known, a meeting was held on February 10 at the rooms of the League of Remembrance, in Marlborough Gate, for the League of Remembrance, besides doing its own good work for the widows of officers, acts as a centre for all forms of help and goodwill to members of the Services. Illness kept Sir Frederick Milner from presiding as he had promised, but his place was taken by Mr. John Murray, and an account of the work was given by Dr. Davis Waite and Dr. Laughton Scott. Each illustrated the need of help by instances from his experience. Dr. Waite told of a man who showed the greatest terror at the sight of a square-shaped loaf of bread. For a long time the cause of this strange fear could not be discovered, but finally it transpired that once at the Front the man was in the act of cutting up a square loaf to share with a comrade by his side when a shell came and blew his friend to pieces. The mental impression renewed itself whenever he saw a similar loaf; but once the cause of the obsession was known it was possible to treat the case. Dr. Scott told the sad case of a medical man who, having suffered from shell-shock in France, was offered when he came home a post in an asylum. Contrary to Dr. Scott's advice, he accepted it, with the result that he is now a patient in the institution. Such cases indicate the need for homes where, in suitable surroundings, the resources of science, aided by patience and kindly care, may be brought to bear upon the sufferers. Electric treatment of all kinds will be provided, with baths of different kinds, careful dieting, and, not least important, all that can be done by psycho-therapy. The Presidents of the Society are Earl Beatty, Earl Haig, and Air-Marshal Sir H. M. Trenchard—men who know best what the men of the Services they represent have had to undergo. But even those who know only by hearsay must, if they have any imagination and any sympathy, pity the men whose nerves have been so shaken in the cause of freedom and honour, and will surely support the Ex-Services Welfare Society in every possible way. The Treasurer is Mrs. Waddingham, and the bankers Lloyds Bank, Piccadilly branch; whilst the offices are at The League of Remembrance, 1 Marlborough Gate, W. 2.

